# Distant homologs of anti-apoptotic factor HAX1 encode parvalbumin-like calcium binding proteins

**DOI:** 10.1186/1756-0500-3-197

**Published:** 2010-07-15

**Authors:** Katarzyna Kokoszyńska, Leszek Rychlewski, Lucjan S Wyrwicz

**Affiliations:** 1 Maria Sklodowska-Curie Memorial Cancer Center and Institute of Oncology, Roentgena 5, 02-781 Warsaw, Poland; 2 BioInfoBank Institute, Limanowskiego 24A, 60-744 Poznań, Poland

## Abstract

**Background:**

Apoptosis is a highly ordered and orchestrated multiphase process controlled by the numerous cellular and extra-cellular signals, which executes the programmed cell death *via *release of cytochrome c alterations in calcium signaling, caspase-dependent limited proteolysis and DNA fragmentation. Besides the general modifiers of apoptosis, several tissue-specific regulators of this process were identified including HAX1 (HS-1 associated protein X-1) - an anti-apoptotic factor active in myeloid cells. Although HAX1 was the subject of various experimental studies, the mechanisms of its action and a functional link connected with the regulation of apoptosis still remains highly speculative.

**Findings:**

Here we provide the data which suggests that HAX1 may act as a regulator or as a sensor of calcium. On the basis of iterative similarity searches, we identified a set of distant homologs of HAX1 in insects. The applied fold recognition protocol gives us strong evidence that the distant insects' homologs of HAX1 are novel parvalbumin-like calcium binding proteins. Although the whole three EF-hands fold is not preserved in vertebrate our analysis suggests that there is an existence of a potential single EF-hand calcium binding site in HAX1. The molecular mechanism of its action remains to be identified, but the risen hypothesis easily translates into previously reported lines of various data on the HAX1 biology as well as, provides us a direct link to the regulation of apoptosis. Moreover, we also report that other family of myeloid specific apoptosis regulators - myeloid leukemia factors (MLF1, MLF2) share the homologous C-terminal domain and taxonomic distribution with HAX1.

**Conclusions:**

Performed structural and active sites analyses gave new insights into mechanisms of HAX1 and MLF families in apoptosis process and suggested possible role of HAX1 in calcium-binding, still the analyses require further experimental verification.

## Background

Apoptosis - the programmed cell death - is one of the basic processes in regulation of cell count in ontogenesis. Early models defined apoptosis as a relatively simple a few step process starting from the initiation by one of pro-apoptotic signals and driving to the final endonucleolytic cleavage of genomic DNA and membrane blebbing. The identification of novel molecular mechanisms involved in this process suggests that the overall picture is far more complex and that the current cell status is directly related to the balance between pro- and anti-apoptotic factors. The induction of apoptosis may be caused by a number of events including disruption of genome, oncogenes' activation, stimulation of various receptors, disorders in calcium homeostasis, free radicals or other damaging factors like radiation [1,2]. Finally, the limited number of apoptosis execution mechanisms is modulated by a wide range of signals [1].

The cell survival depends on homeostasis of signals involved in induction and inhibition of both proliferation and apoptosis. Within a set of regulators of apoptosis, several key proteins were already identified (i.e. Bcl-2, IAP, survivin) [3]. Two of the proteins constitute myeloid regulators of apoptosis: HS-1- associated protein X-1 (HAX1) and myeloid leukemia factors (MLF1, MLF2). Although these proteins were the subjects of several experimental studies, mechanisms of their activity remain to be only partially discovered.

HS-1 associated protein X-1 (HAX1), is a multifunctional protein first identified in a yeast two hybrid system as a partner of HS-1 (hematopoietic lineage cell-specific protein I) [4]. So far, HAX1 has been detected in various cellular compartments and is known to be involved in a number of different processes. The high level of its expression is observed in mitochondrion and nuclear matrix, although HAX1 was also found in endoplasmic reticulum, apical membrane of hepatocytes and nuclear envelope [5]. *HAX1 *is expressed ubiquitously among various tissues, mainly in skeletal muscle and heart [6] and in a number of cancer tissues [7,8]. Although HAX1 is known as a multifunctional protein, the exact mechanism of its action remains unexplained [9].

Currently, there are several observations that are linking HAX1 and cell apoptosis together. As reported by Vafiadaki *et al. *a putative HAX1's anti-apoptotic role may result from an inhibition of caspase 9. HAX1 repress post-mitochondrial caspase 9 activation, cell death during hypoxia and following re-oxygenation and its overexpression protects cardiac myocytes from apoptosis [10]. Here, the C-terminal part of HAX1 interacts with caspase-9 at the region corresponding to residues 175-206 of human ortholog (GenBank identifier - gi|158562115), even though, both N-terminal and C-terminal domains are required for full anti-apoptotic function [11,12]. Additionally, HAX1 interacts with other partners from the caspase cascade. Caspase 3 (CASP3) cleaves HAX1 at residue Asp127, while HAX1 over-expression inhibits CASP3 catalytic activity and blocks the initiation of apoptosis [13].

**Figure 1:**
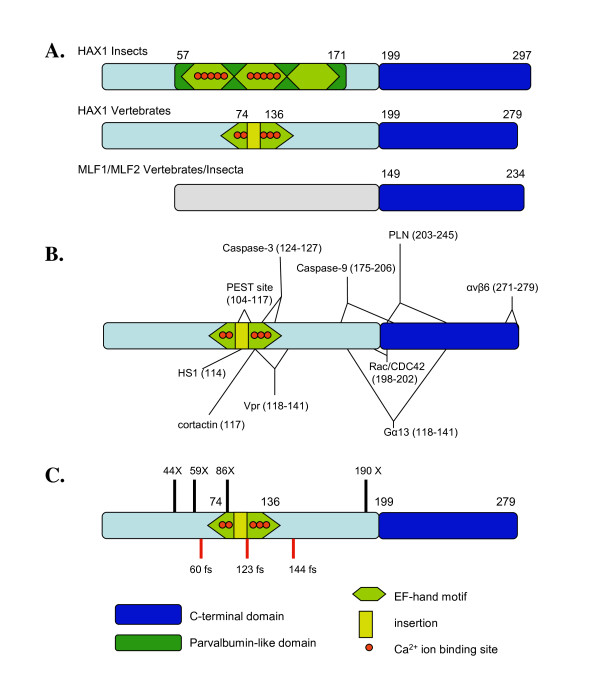
**A. The overview of the domain composition of HAX1 and MLF proteins**. **B**. Interaction sites within human HAX1 protein. **C**. Mutation sites within HAX1 (X-nonsense mutation, fs - frameshift).

Several previous studies suggested HAX1 involvement in calcium homeostasis [6,10]. Phospholamban (PLN) - a transmembrane regulator of the contractility in the heart and calcium homeostasis - binds to HAX1 within internal part of protein (residues 203-245 of human ortholog - gi|158562115, compare Figure 1B). The HAX1-PLN complex plays an important role in cardiac cell survival and the presence of PLN enhances the anti-apoptotic potential of HAX1. PLN regulates activity of the sarcoplasmic reticulum Ca^2+ ^- ATP-ase pump (SERCA2a) - the regulator of heart calcium homeostasis [6]. Formation of the HAX1-PLN complex is modulated and interaction with HAX1 is reduced by either phosphorylation of PLN or increased concentration of Ca^2+ ^[6]. Additionally, HAX1 when bound to PLN is redistributed from mitochondrion to endoplasmatic reticulum (ER) [6]. ER localization is also crucial for interaction of HAX1 with polycystic kidney disease 2 (PKD2) apoptosis regulating protein involved in Ca^2+ ^signaling in kidney cells. Here, HAX1 acts as an adaptor between PKD2 and cortactin - a key regulator of PKD2 function [14].

HAX1 was previously described as a hairpin-structure RNA binding protein [15]. This observation was initially raised on the basis of interaction with vimentin's mRNA 3'UTR [15]. Sarnowska *et al. *suggested also that HAX1 plays a role in destabilization of mRNA through binding of Polβ (polymerase beta) mRNA.

Since multifunctional activity and a number of interactions involved mainly in regulation of apoptosis are the key processes in cell regulation, this protein plays important role in several diseases and carcinogenesis. One of the most important functions of HAX1 is the regulation of myeloid homeostasis. This protein is crucial for proper inner mitochondrial membrane potential and for protecting myeloid cells from apoptosis [16,17]. Congenital deficiency of HAX1, leads to the development of Kostmann syndrome [17], *via *mitochondrion-dependent deregulation of apoptosis, which results in an infantile genetic agranulocytosis. Notable, in the later phase Kostmann syndrome can lead to the development of acute myeloid leukemia [17].

The data on the activity of myeloid leukemia factor 1 (MLF1) is far more limited. At first, *MLF1 *was identified in an oncogenic fusion gene with nucleophosmin (*NPM-MLF1*). This fusion gene is generated by t(3;5)(q25.1;q34) translocation [18-20] and is linked with both acute myeloid leukemia (AML) and myelodysplastic syndrome (MDS). *MLF1 *is expressed in a number of tissues, including skeletal muscle, heart, brain and hematopoietic stem cells. Despite several reports about MLF family, mechanisms of its activity have not been reported. The previous analyses contain quite inconsistent data suggesting variable range of its actions. *MLF1 *protein product is mainly targeted to cytoplasm and is potentially involved in regulation of mitosis (cell cycle exit) and cell differentiation processes [19,21]. Several lines of evidence suggest that MLF1 also participates in induction of apoptosis when fused with NPM [20,22]. MLF1 - as a component of NPM-MLF1 complex - is known to be involved in various pathways of carcinogenesis. It negatively regulates the progression of cell cycle inhibiting cell growth through the accumulation of tumor suppressor protein p53 (TP53) [23]. Additionally, MLF1 in hemopoetic lineage is able to switch erythroleukemic cells to the monoblastoid phenotype. It can also deregulate pluripotent progenitor cells, participate in ineffective hematopoiesis and transformation into leukemia [20].

Similarly to *MLF1*, the chromosomal translocations and deletions of myeloid leukemia factor 2 (*MLF2*) locus were also observed in acute leukemia [24], but no direct studies were performed to identify the specific mechanism of its action. Both proteins share 64% of sequence similarity between human homologs (gi|194328680 and gi|54695734, MLF1 and MLF2 respectively), but no experimental studies on MLF2 were published recently. The lack of data on MLFs is reflected in the major secondary database - Sanger's Protein Families (Pfam). Although sequences of MLF1 (Q2TLE5_MLF1_HUMAN) and MLF2 (Q15773_MLF2_HUMAN) were deposited under the PfamA accession PF10248, the title of the entry as well as the description of the domain, are related to non-homologous protein MLF1-interacting protein (MLF1IP) (Q71F23|CENPU_HUMAN), what shows a misleading assignment of the MLF proteins. Comparison of MLF and MLF1IP proteins shoved only marginal similarity score (expected value above 2).

Identification of various pro- and anti-apoptotic pathways is a challenging problem. So far, no significant homology of the two discussed protein families connected to other proteins of similar function was reported. Several previous studies described inconsistent data on the structure of HAX1. Sharp *et al. *postulated that there is a presence of a transmembrane helix in the C-terminal region of open reading frame (position 261-273) [25], while other researchers suggested that, the motif is shorter than an ordinary transmembrane helix [4]. Suzuki *et al. *reported that the N-terminal region of HAX1 shows weak similarity to NIP3 protein which is up-regulated in oxidative stress conditions in cardiac myocytes. It is also involved in apoptosis which causes mitochondrial defects [26]. Another two regions consisting of residues 37- 56 and 74-89 of human HAX1 is reported to be homologous to anti-apoptotic protein, therefore, BCL2 (B-cell lymphoma 2) creating so called BCL2-homology domains, respectively BH1 and BH2 [11,16,26]. The detailed revision of the presented bioinformatic evidence reported in the above cited articles shows that original reports contain inappropriate application of bioinformatic methods and could not be reproduced. To summarize, as of now, no clear data on homology of either HAX1 or MLFs has previously been reported.

Previously we were able to successfully apply bioinformatic methods of fold recognition and identification of distant homology to analyze genes participating in important biological processes. These include ALK kinase regulator *NIPA *[3], family of transcription factors homologous to *TFCP2 *regulating transcription of globins [27] and several other human and viral genes [28,29]. In this work we present a bioinformatic approach to elucidate the molecular function of *HAX1 *and *MLF1 *homologs.

## Methods

Sequences of HAX1 and MLF1 proteins homologs were collected using PSI-BLAST (default settings) [30] in NR (non-redundant) database of NCBI (National Center for Biotechnology Information; http://www.ncbi.nlm.nih.gov). The sequences of each protein were clustered at 70% of sequence identity with CD-HIT [31] in order to select representative collection of protein sequences, with preserved functional motifs. The final protein sequence collections were aligned with ClustalW [32] and PCMA [33]. Based on the alignment conserved regions were selected. Conserved sequence regions of both families were subjected to iterative PSI-BLAST searches at NCBI resources. Sequences from each protein family were submitted to the Protein Structure Prediction MetaServer (http://bioinfo.pl/meta; [34]) - server joining a number of secondary structure prediction (PsiPred [35]), fold recognition (3D-PSSM [36], INUB [37], SAM_T06 [38]) and homology modeling methods (FFAS03 [39], MetaBasic [40]). Based on secondary structure predictions, location of critical residues and physicochemical properties of amino acids multiple sequence alignment of HAX1 and MLF protein families was manually corrected. Protein homology models of HAX1 parvalbumin-like domain were calculated with Modeler v. 9 based on 1RJP PDB structure [41] and implemented in Discovery Studio 2.1 (Accelerys Inc.). Visualizations of the obtained structures were performed with PyMol http://pymol.sourceforge.net/.

## Results and discussion

### Identification of protein domains

**Figure 2:**
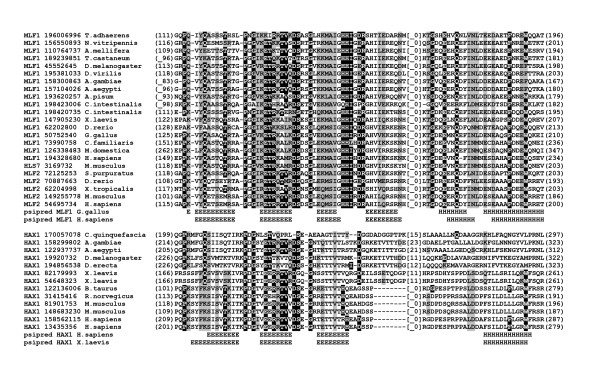
**Multiple sequence alignment of HAX1 and MLF C-terminal domains**. The corresponding sequences were defined with GenBank entries (coded with GenBank identifier - gi) and origin organism. Numbers in brackets refer to the positions of presented sequence fragments, numbers in square brackets indicate the number of residues removed for clarity of alignment. Predicted secondary structure elements (Psipred) are coded with letters (H - α-helix, E - extended).

Based on sequence searches for the whole length of HAX1 and MLFs no significant similarity in NR database (NCBI) was detected. Similar searches applied only with the most conserved C-terminal part of HAX1 as a query, revealed a significant match to C-terminal domain of Myeloid Leukemia Factor family (Figure 1A). The comparison of similar regions of both protein families, together with the results of secondary structure prediction, confirmed the initial homology searches. The secondary structure prediction analysis performed for both protein families revealed a common pattern of secondary structure elements (three beta strands and an alpha helix at the carboxyl terminus of the proteins; Figure 2). The conserved domain is localized at positions 209-287, 149-234, 115-200 of human HAX1 (gi|158562115), MLF1 (gi|194328680) and MLF2 (gi|54695734) respectively. The most conserved region used as an initial query, spanned the potential β-sheet. The last common α-helix is preceded by an additional strand and marginal prediction of α-helix in MLF family. The final multiple sequence alignment of C-terminal parts of HAX1 and MLF protein families shown in Figure 2 was created by manual correction of the obtained multiple sequence alignment (ClustalW, PCMA).

The high conservation of C-terminal domain of these proteins suggest its importance in protein's function of apoptosis regulation. Within C-terminal regions of the two protein families, several similarities can be observed. As shown in Figure 2, common distribution of the secondary structure elements, a high conservation of several key residues and properties of corresponding amino acids supports their homology and suggest a common fold for these two protein families. Preserved distribution of charged and hydrophobic amino acids, presence of characteristic glycine residues between β-sheets of both protein families point to similarity of the predicted domain structure. Detailed analyses revealed no assignments to any known records in protein families databases (Pfam, SMART).

Although a number of protein structure prediction methods were applied via the Protein Structure Prediction MetaServer, no significant similarity to known protein structures was detected, indicating the probable new fold type. Since no proteins of homologous experimentally determined structure were found, fold recognition for HAX1 and MLFs C-terminal domain could not be performed.

The analysis of taxonomic distribution of homologous sequences of both families revealed additional similarities of these two protein families - both HAX1 and MLFs families are well conserved through *Vertebrata*, but surprisingly additional homologues of both families were found in genomes of insects.

### Fold recognition of the N-terminal calcium binding domain

Notably, HAX1 homologs in insects are less divergent of remaining sequences of the whole protein family (whole sequence group exhibiting homology and likely to perform similar function). The application of several threading and profile-profile methods via Protein Structure Prediction MetaServer for fragments of insects HAX1 sequences showed marginal similarity scores to parvalbumin's EF-hand calcium binding motifs in the N-terminal region of HAX1 (gi|122937737:57-171 - supported by Meta Basic, INUB and 3D-PSSM; 3D-Jury score: 60.20).

Parvalbumin belongs to the superfamily of calcium binding proteins [42]. The overall function of this family is related to transport and storage of calcium ions. Present mainly in muscle and neurons; it is responsible for muscle relaxation and calcium transport from myofibrils to the sarcoplasmic reticulum. It may also affect some cellular properties such as: extension of the G1 cell cycle phase, increase of cell motility and extension of mitotic rate in ovarian carcinoma calls [42]. From the structural point of view, parvalbumin is composed of six α-helices forming three helix-loop-helix motifs known as EF-hand motifs. Hydrophobic surfaces of the helices form a core part of this fold. These motifs between two adjacent amphipathic helices contain a loop of different length coordinating calcium ions mainly by carboxylate groups of aspartic and glutamic acid side chains. The six helices form three pairs - the acidic residues (Asp, Glu) at loops between the second and the third pair of helices are involved in binding of ions. The loop between first helix pair, because of the absence of critical amino acids is unable to bind calcium ions [43,44].

**Figure 3:**
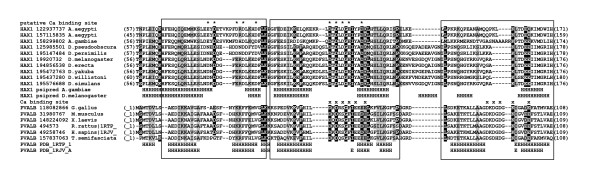
**Multiple sequence alignment of insects HAX1 and Parvalbumin (PVALB) calcium binding domains**. The corresponding sequences were defined with GenBank entries (coded with GenBank identifier - gi) and origin organism. Numbers in brackets refer to the positions of presented sequence fragments. The observed (Protein Data Bank entry 1RTP_1, 1RJV_A) and predicted (Psipred) secondary structure elements are coded with letters (H - α-helix,E - β-strands). Calcium binding sites within parvalbumin proteins are marked with x, putative calcium binding residues of HAX1 are marked with asterisks (*).

The manual adjustment of multiple sequence alignment of *Insecta *HAX1 with parvalbumin sequences confirms the overall similarity of these two protein families. The final alignment of HAX1 sequences of insects with imposition of EF-hand motif is shown in Figure 3. The prediction of secondary structure of HAX1 was highly consistent with observed six helices of parvalbumin (three consecutive EF-hand motifs). The analysis of biochemical properties of aligned residues pointed to the conserved pattern of hydrophobic surfaces of helices building the fold. Surprisingly, the presence of acidic residues that are critical for calcium ions binding within HAX1 sequences suggested that the first and second EF-hand motifs are involved in forming of calcium binding sites, while in parvalbumin, such motifs are located within second and third motif [43,44]. The acidic residues within the third motif, located between fifth and sixth helix, are in the fact not preserved in instects' HAX1. Visualization of HAX1 models revealed that only within the second EF-hand motif of HAX1, the location of critical amino acids involved in formation of binding site can analogously to typical EF-hand motif (typically for parvalbumin) bind calcium ions. The role of potential site within first EF-hand motif remains highly speculative (Additional file 1).

**Figure 4:**
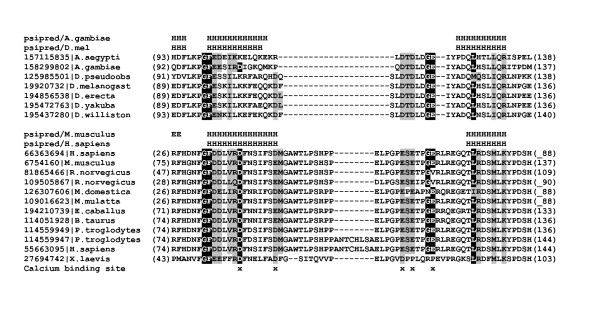
**Multiple sequence alignment of insects and vertebrates HAX1 proteins putative calcium binding motifs**. The corresponding sequences were defined with GenBank entries (coded with GenBank identifier - gi) and origin organism. Numbers in brackets refer to the positions of presented sequence fragments. Predicted secondary structure elements (Psipred) are coded with letters (H - α-helix,E - β-strands). Putative calcium binding residues of vertebrates HAX1 are marked with x.

The fold recognition for insects' HAX1 gives a strong suggestion that this protein initially evolved as a regulator of calcium homeostasis. When compared to insect HAX1 proteins only a single EF-hand motif consisting of two alpha-helices is preserved in vertebrate HAX1 proteins. The detailed alignment of the HAX1 proteins of vertebrates with recognized calcium binding motif of insects' suggested the presence of a single active calcium binding site (Figure 3). This motif was preserved throughout the whole clade (*Insecta*) and the presence of hydrophobic residues within helices along with pattern of acidic binding site within loops. However, the active site in vertebrates HAX1 proteins diverged from typical EF-hand motif with additional 20-28 amino acids within the calcium binding loop region (Figure 4). Furthermore, the precise confirmation of the motif's functionality requires detailed analyses. The sequence conservation and secondary structure predictions failed to identify any known potential functional motif. Previous studies suggested that EF-hand remains functional only in multimeric composition [45]. Although within vertebrates' HAX1, we identified only a single motif corresponding to the classic three EF-hand domain our prediction can still be correct since calcium ion binding can occur by forming a dual EF-hand domain composition during formation of HAX1 dimers [46]. Summarizing, in the current state of data, the presence of active EF-hand motif in vertebrates' HAX1 is still speculative and detailed experimental studies are needed to confirm this hypothesis.

### Location of HAX1 interaction sites

According to the current state of the knowledge, specific function of HAX1 is a final result of its various interactions. At first HAX1 was identified as a binding partner of HS1 (hematopoietic lineage cell-specific protein I), a substrate of Src family tyrosine kinase. HS1 protein is expressed specifically in hematopoietic cells and implicated in signal transduction in B cells. The complex formation proceeds between N-terminal part of HS1 (residues 27-66) and C-terminal part of HAX1. This probably results in altered signal transduction from the Ag receptor (B lymphocyte antigen receptor) to intracellular organelles. When associated with HAX1, HS1 is accumulated in mitochondrial membrane [26]. Beside this, a number of other interactions with cellular and viral proteins has been described [25,47-51]. Such a broad spectrum of interacting proteins of unrelated structures suggests that HAX1 is a protein involved in intracellular signaling and is harboring various intracellular molecules (hub protein). Most of the known interaction sites within HAX1 are located among two regions defined previously as putative functional domains (all the known interaction sites are shown on Figure 1B). This further supports the hypothesis of two functional domains of HAX1 spaced by additional unstructured protein-binding region.

### Apoptosis regulation

Several studies indicated that HAX1 is involved in apoptosis regulation [10,13]. Previous analyses linked apoptosis event with interactions of HAX1 with proteins of caspase cascade (caspase 3 and 9). Both N-terminal and C-terminal domains are required for full anti-apoptotic function [11,12]. Caspase 3 interacts with a region within predicted calcium binding site (cleaves HAX1 at residue Asp127). HAX1 when over-expressed, inhibits CASP3 catalytic activity and blocks the initiation of apoptosis [13]. C-terminal part of HAX1 interacts with caspase-9 at the region corresponding to residues 175-206 of human ortholog (gi|158562115). HAX1 repress post-mitochondrial caspase 9 activation and cell death during hypoxia-reoxygenation and HAX1 overexpression protects cardiac myocytes from apoptosis [10]. Location of HAX1 interaction sites with both caspases within functional regions described in this report partially supports the involvement of both of these regions in apoptosis and proper function of HAX1.

Additionally, several points and frame shift mutations within *HAX1 *were previously described. This genetic alterations associated with neutrophil depletion [52] in fact affect mainly functional regions described here (compare Figure 1C).

### RNA binding properties of HAX1

The previous data reports that mRNA binding occurred within C-terminal part of the protein only in a diametric form of HAX1. The overall picture of mechanism of HAX1 binding to RNA is still not clear. According to the results of assays performed with HAX1 deletion mutants, a potential nucleic acid binding region is located within carboxyl terminus of HAX1.

According to predictions of secondary structure - the C-terminal region contains three conserved fragments which seem to be in extended conformation, likely to form a beta-sheet. This local conformation is conserved also in MLFs. Although some RNA-binding folds consist mainly of beta-sheet, our analyzes did not allow to map a distant similarity of HAX1 or MLFs to any known RNA-binding proteins. There are two major explanations for this situation: either HAX1 forms a novel RNA-binding protein, or this C-terminal fragment forms rather a platform closing together other protein agents, where some possess RNA binding activity [9].

## Conclusions

Although the mechanisms of HAX1 and MLF1 activity are not well described, the applied state-of-the-art of protein structural bioinformatics revealed several common traces of their biology. In the history of the molecular evolution of HAX1-MLF1 families we can indicate three distinct points.

The C-terminal domain of HAX1-MLF1 families revealed a sequence homology which can suggest the existence of novel protein structural domain potentially involved in RNA (or other protein) binding.

Similar taxonomic distribution of homologous sequences and conservation of C-terminal domain of both families emphasize its importance in proteins common function of apoptosis regulation. It is worth to note that we made an interesting observation that HAX1 and MLFs are present only in co-existence in a number of genomes. This might suggest that there is either physical interaction between their protein products or that there is a novel negative feed-back mechanism in regulation of related biological processes.

Additionally, N-terminal domain of HAX1 family was described. HAX1 homologues in insects showed marginal similarity to parvalbumin EF-hand calcium binding motifs with the second EF-hand motif pointed as a calcium binding site. In vertebrates' HAX1 - only a single EF-hand motif consisting of two α-helices with additional 20-28 amino acids within the calcium binding loop region is preserved. Performed structural and active sites analyses gave new insights into mechanisms of HAX1 and MLF families in apoptosis process and suggested possible role of HAX1 in calcium-binding, still the analyses require further experimental verification.

In the presented report we describe the application of several methods of homology search and protein structure prediction in drafting of hypotheses on function of HAX1. In the light of this new data, additional experimental work is needed to confirm and summarize a picture of HAX1 and MLFs biology.

## Competing interests

The authors declare that they have no competing interests.

## Authors' contributions

KK, LSW participated in the design of the study. KK, LR and LSW carried out the bioinformatics analysis. KK, LSW wrote the manuscript. All authors read and approved the final manuscript.

## Supplementary Material

Additional file 1 **Model of *Aedes aegypti *HAX1 parvalbumin-like domain**. Putative two EF-hand calcium binding site of *Aedes aegypti *HAX1 parvalbumin-like domain modeled on the template of parvalbumin (PDB: 1RJP). A. N-terminal EF-hand motif - critical residues marked in blue; B. C-terminal EF-hand motif - critical residues likely to be involved in formation of calcium binding module were shown in red. Click here for file

## References

[B1] Reed JC Doctor KS Godzik A The domains of apoptosis: a genomics perspective Sci STKE 2004 2004 re9 10.1126/stke.2392004re9 15226512

[B2] Schultz DR Harrington WJ Jr Apoptosis: programmed cell death at a molecular level Semin Arthritis Rheum 2003 32 345 369 10.1053/sarh.2003.50005 12833244

[B3] Kokoszynska K Rychlewski L Wyrwicz LS The mitotic entry regulator NIPA is a prototypic BIR domain protein Cell Cycle 2008 7 2073 2075 18604162 10.4161/cc.7.13.6237

[B4] Ortiz DF Moseley J Calderon G Swift AL Li S Arias IM Identification of HAX-1 as a protein that binds bile salt export protein and regulates its abundance in the apical membrane of Madin-Darby canine kidney cells J Biol Chem 2004 279 32761 32770 10.1074/jbc.M404337200 15159385

[B5] Sarnowska E Grzybowska EA Sobczak K Konopinski R Wilczynska A Szwarc M Sarnowski TJ Krzyzosiak WJ Siedlecki JA Hairpin structure within the 3'UTR of DNA polymerase beta mRNA acts as a post-transcriptional regulatory element and interacts with Hax-1 Nucleic Acids Res 2007 35 5499 5510 10.1093/nar/gkm502 17704138 PMC2018635

[B6] Vafiadaki E Sanoudou D Arvanitis DA Catino DH Kranias EGA Kontrogianni-Konstantopoulos, Phospholamban interacts with HAX-1, a mitochondrial protein with anti-apoptotic function J Mol Biol 2007 367 65 79 10.1016/j.jmb.2006.10.057 17241641

[B7] Ramsay AG Keppler MD Jazayeri M Thomas GJ Parsons M Violette S Weinreb P Hart IR Marshall JF HS1-associated protein X-1 regulates carcinoma cell migration and invasion via clathrin-mediated endocytosis of integrin alphavbeta6 Cancer Res 2007 67 5275 5284 10.1158/0008-5472.CAN-07-0318 17545607

[B8] Mirmohammadsadegh A Tartler U Michel G Baer A Walz M Wolf R Ruzicka T Hengge UR HAX-1, identified by differential display reverse transcription polymerase chain reaction, is overexpressed in lesional psoriasis J Invest Dermatol 2003 120 1045 1051 10.1046/j.1523-1747.2003.12247.x 12787133

[B9] Fadeel B Grzybowska E HAX-1: a multifunctional protein with emerging roles in human disease Biochim Biophys Acta 2009 19524642 10.1016/j.bbagen.2009.06.004

[B10] Vafiadaki E Papalouka V Arvanitis DA Kranias EG Sanoudou D The role of SERCA2a/PLN complex, Ca(2+) homeostasis, and anti-apoptotic proteins in determining cell fate Pflugers Arch 2009 457 687 700 10.1007/s00424-008-0506-5 18415121

[B11] Shaw J Kirshenbaum LA HAX-1 represses postmitochondrial caspase-9 activation and cell death during hypoxia-reoxygenation Circ Res 2006 99 336 338 10.1161/01.RES.0000239408.03169.94 16917098

[B12] Han Y Chen YS Liu Z Bodyak N Rigor D Bisping E Pu WT Kang PM Overexpression of HAX-1 protects cardiac myocytes from apoptosis through caspase-9 inhibition Circ Res 2006 99 415 423 10.1161/01.RES.0000237387.05259.a5 16857965

[B13] Lee AY Lee Y Park YK Bae KH Cho S Lee do H Park BC Kang S Park SG HS 1-associated protein X-1 is cleaved by caspase-3 during apoptosis Mol Cells 2008 25 86 90 18319618

[B14] Gallagher AR Cedzich A Gretz N Somlo S Witzgall R The polycystic kidney disease protein PKD2 interacts with Hax-1, a protein associated with the actin cytoskeleton Proc Natl Acad Sci USA 2000 97 4017 4022 10.1073/pnas.97.8.4017 10760273 PMC18134

[B15] Al-Maghrebi M Brule H Padkina M Allen C Holmes WM Zehner ZE The 3' untranslated region of human vimentin mRNA interacts with protein complexes containing eEF-1gamma and HAX-1 Nucleic Acids Res 2002 30 5017 5028 10.1093/nar/gkf656 12466525 PMC137969

[B16] Grzybowska EA Sarnowska E Konopinski R Wilczynska A Sarnowski TJ Siedlecki JA Identification and expression analysis of alternative splice variants of the rat Hax-1 gene Gene 2006 371 84 92 10.1016/j.gene.2005.11.035 16516414

[B17] Carlsson G Melin M Dahl N Ramme KG Nordenskjold M Palmblad J Henter JI Fadeel B Kostmann syndrome or infantile genetic agranulocytosis, part two: Understanding the underlying genetic defects in severe congenital neutropenia Acta Paediatr 2007 96 813 819 10.1111/j.1651-2227.2007.00274.x 17537008

[B18] Lim R Winteringham LN Williams JH McCulloch RK Ingley E Tiao JY Lalonde JP Tsai S Tilbrook PA Sun Y Wu X Morris SW Klinken SP MADM, a novel adaptor protein that mediates phosphorylation of the 14-3-3 binding site of myeloid leukemia factor 1 J Biol Chem 2002 277 40997 41008 10.1074/jbc.M206041200 12176995

[B19] Li ZF Wu X Jiang Y Liu J Wu C Inagaki M Izawa I Mizisin AP Engvall E Shelton GD Non-pathogenic protein aggregates in skeletal muscle in MLF1 transgenic mice J Neurol Sci 2008 264 77 86 10.1016/j.jns.2007.07.027 17854834

[B20] Yoneda-Kato N Fukuhara S Kato J Apoptosis induced by the myelodysplastic syndrome-associated NPM-MLF1 chimeric protein Oncogene 1999 18 3716 3724 10.1038/sj.onc.1202711 10391679

[B21] Winteringham LN Endersby R Kobelke S McCulloch RK Williams JH Stillitano J Cornwall SM Ingley E Klinken SP Myeloid leukemia factor 1 associates with a novel heterogeneous nuclear ribonucleoprotein U-like molecule J Biol Chem 2006 281 38791 38800 10.1074/jbc.M605401200 17008314

[B22] Matsumoto N Yoneda-Kato N Iguchi T Kishimoto Y Kyo T Sawada H Tatsumi ES Fukuhara, Elevated MLF1 expression correlates with malignant progression from myelodysplastic syndrome Leukemia 2000 14 1757 1765 10.1038/sj.leu.2401897 11021751

[B23] Yoneda-Kato N Kato JY Shuttling imbalance of MLF1 results in p53 instability and increases susceptibility to oncogenic transformation Mol Cell Biol 2008 28 422 434 10.1128/MCB.02335-06 17967869 PMC2223285

[B24] Kuefer MU Look AT Williams DC Valentine V Naeve CW Behm FG Mullersman JE Yoneda-Kato N Montgomery K Kucherlapati R Morris SW cDNA cloning, tissue distribution, and chromosomal localization of myelodysplasia/myeloid leukemia factor 2 (MLF2) Genomics 1996 35 392 396 10.1006/geno.1996.0376 8661158

[B25] Sharp TV Wang HW Koumi A Hollyman D Endo Y Ye H Du MQ Boshoff C K15 protein of Kaposi's sarcoma-associated herpesvirus is latently expressed and binds to HAX-1, a protein with antiapoptotic function J Virol 2002 76 802 816 10.1128/JVI.76.2.802-816.2002 11752170 PMC136811

[B26] Suzuki Y Demoliere C Kitamura D Takeshita H Deuschle U Watanabe T HAX-1, a novel intracellular protein, localized on mitochondria, directly associates with HS1, a substrate of Src family tyrosine kinases J Immunol 1997 158 2736 2744 9058808

[B27] Kokoszynska K Ostrowski J Rychlewski L Wyrwicz LS The fold recognition of CP2 transcription factors gives new insights into the function and evolution of tumor suppressor protein p53 Cell Cycle 2008 7 2907 2915 18787404 10.4161/cc.7.18.6680

[B28] Wyrwicz LS Ginalski K Rychlewski L HSV-1 UL45 encodes a carbohydrate binding C-type lectin protein Cell Cycle 2008 7 269 271 18256535 10.4161/cc.7.2.5324

[B29] Knizewski L Steczkiewicz K Kuchta K Wyrwicz L Plewczynski D Kolinski A Rychlewski L Ginalski K Uncharacterized DUF1574 leptospira proteins are SGNH hydrolases Cell Cycle 2008 7 542 544 18235229 10.4161/cc.7.4.5386

[B30] Altschul SF Madden TL Schaffer AA Zhang J Zhang Z Miller WDJ Lipman, Gapped BLAST and PSI-BLAST: a new generation of protein database search programs Nucleic Acids Res 1997 25 3389 3402 10.1093/nar/25.17.3389 9254694 PMC146917

[B31] Li W Godzik A Cd-hit: a fast program for clustering and comparing large sets of protein or nucleotide sequences Bioinformatics 2006 22 1658 1659 10.1093/bioinformatics/btl158 16731699

[B32] Thompson JD Gibson TJ Higgins DG Multiple sequence alignment using ClustalW and ClustalX Curr Protoc Bioinformatics 2002 Chapter 2 Unit 2 3 18792934 10.1002/0471250953.bi0203s00

[B33] Pei J Sadreyev R Grishin NV PCMA: fast and accurate multiple sequence alignment based on profile consistency Bioinformatics 2003 19 427 428 10.1093/bioinformatics/btg008 12584134

[B34] Bujnicki JM Elofsson A Fischer D Rychlewski L Structure prediction meta server Bioinformatics 2001 17 750 751 10.1093/bioinformatics/17.8.750 11524381

[B35] McGuffin LJ Bryson K Jones DT The PSIPRED protein structure prediction server Bioinformatics 2000 16 404 405 10.1093/bioinformatics/16.4.404 10869041

[B36] Kelley LA MacCallum RM Sternberg MJ Enhanced genome annotation using structural profiles in the program 3D-PSSM J Mol Biol 2000 299 499 520 10.1006/jmbi.2000.3741 10860755

[B37] Fischer D 3D-SHOTGUN: a novel, cooperative, fold-recognition meta-predictor Proteins 2003 51 434 441 10.1002/prot.10357 12696054

[B38] Gao X Bu D Xu J Li M Improving consensus contact prediction via server correlation reduction BMC Struct Biol 2009 9 28 10.1186/1472-6807-9-28 19419562 PMC2689239

[B39] Jaroszewski L Rychlewski L Li Z Li W Godzik A FFAS03: a server for profile--profile sequence alignments Nucleic Acids Res 2005 33 W284 288 10.1093/nar/gki418 15980471 PMC1160179

[B40] Ginalski K von Grotthuss M Grishin NV Rychlewski L Detecting distant homology with Meta-BASIC Nucleic Acids Res 2004 32 W576 581 10.1093/nar/gkh370 15215454 PMC441508

[B41] Eswar N Webb B Marti-Renom MA Madhusudhan MS Eramian D Shen MY Pieper U Sali A Comparative protein structure modeling using MODELLER Curr Protoc Protein Sci 2007 Chapter 2 Unit 2 9 18429317 10.1002/0471140864.ps0209s50

[B42] Pauls TL Cox JA Berchtold MW The Ca2+(-)binding proteins parvalbumin and oncomodulin and their genes: new structural and functional findings Biochim Biophys Acta 1996 1306 39 54 8611623 10.1016/0167-4781(95)00221-9

[B43] McPhalen CA Sielecki AR Santarsiero BD James MN Refined crystal structure of rat parvalbumin, a mammalian alpha-lineage parvalbumin, at 2.0 A resolution J Mol Biol 1994 235 718 732 10.1006/jmbi.1994.1023 8289291

[B44] Baig I Bertini I Del Bianco C Gupta YK Lee YM Luchinat CA Quattrone, Paramagnetism-based refinement strategy for the solution structure of human alpha-parvalbumin Biochemistry 2004 43 5562 5573 10.1021/bi035879k 15122922

[B45] Grabarek Z Structural basis for diversity of the EF-hand calcium-binding proteins J Mol Biol 2006 359 509 525 10.1016/j.jmb.2006.03.066 16678204

[B46] Zhou Y Yang W Kirberger M Lee HW Ayalasomayajula G Yang JJ Prediction of EF-hand calcium-binding proteins and analysis of bacterial EF-hand proteins Proteins 2006 65 643 655 10.1002/prot.21139 16981205

[B47] Cilenti L Soundarapandian MM Kyriazis GA Stratico V Singh S Gupta S Bonventre JV Alnemri ES Zervos AS Regulation of HAX-1 anti-apoptotic protein by Omi/HtrA2 protease during cell death J Biol Chem 2004 279 50295 50301 10.1074/jbc.M406006200 15371414

[B48] Kasashima K Ohta E Kagawa Y Endo H Mitochondrial functions and estrogen receptor-dependent nuclear translocation of pleiotropic human prohibitin 2 J Biol Chem 2006 281 36401 36410 10.1074/jbc.M605260200 17008324

[B49] Kawaguchi Y Nakajima K Igarashi M Morita T Tanaka M Suzuki M Yokoyama A Matsuda G Kato K Kanamori M Hirai K Interaction of Epstein-Barr virus nuclear antigen leader protein (EBNA-LP) with HS1-associated protein X-1: implication of cytoplasmic function of EBNA-LP J Virol 2000 74 10104 10111 10.1128/JVI.74.21.10104-10111.2000 11024139 PMC102049

[B50] Dufva M Olsson M Rymo L Epstein-Barr virus nuclear antigen 5 interacts with HAX-1, a possible component of the B-cell receptor signalling pathway J Gen Virol 2001 82 1581 1587 11413368 10.1099/0022-1317-82-7-1581

[B51] Yedavalli VS Shih HM Chiang YP Lu CY Chang LY Chen MY Chuang CY Dayton AI Jeang KT Huang LM Human immunodeficiency virus type 1 Vpr interacts with antiapoptotic mitochondrial protein HAX-1 J Virol 2005 79 13735 13746 10.1128/JVI.79.21.13735-13746.2005 16227293 PMC1262574

[B52] Smith BN Ancliff PJ Pizzey A Khwaja A Linch DC Gale RE Homozygous HAX1 mutations in severe congenital neutropenia patients with sporadic disease: a novel mutation in two unrelated British kindreds Br J Haematol 2009 144 762 770 10.1111/j.1365-2141.2008.07493.x 19036076

