# Supplementary figures and images for: Distant homologs of anti-apoptotic factor HAX1 encode parvalbumin-like calcium binding proteins
Source: BMC Res Notes. 2010 Jul 15;3:197. doi: 10.1186/1756-0500-3-197 (PMC2914655; doi:10.1186/1756-0500-3-197)

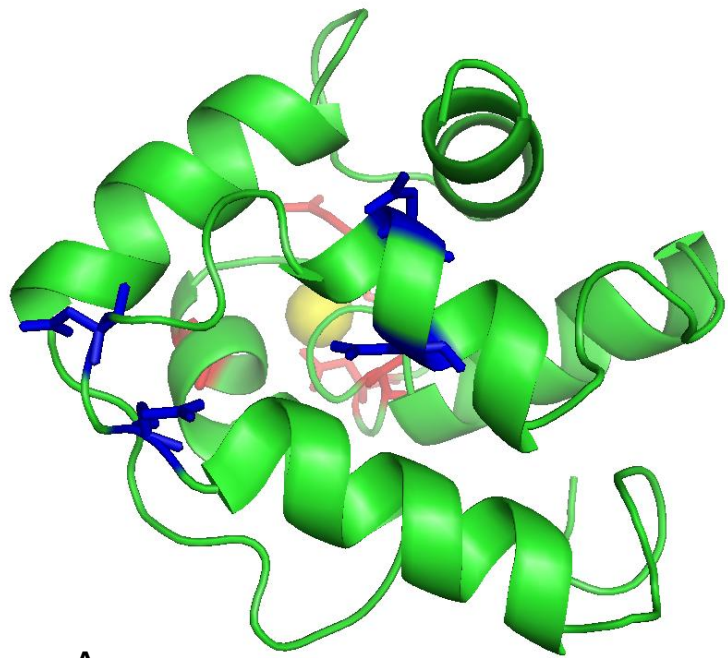

A.

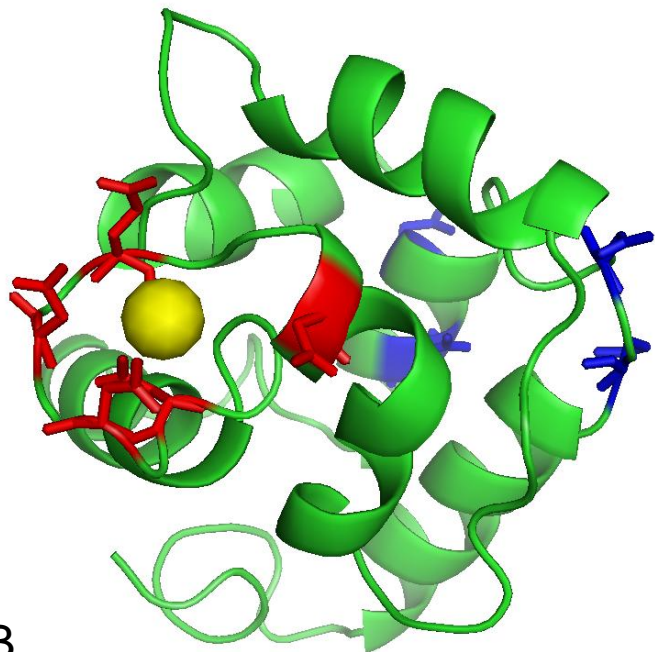

B.

Supplement: Additional file 1 — Model of Aedes aegypti HAX1 parvalbumin-like domain. Putative two EF-hand calcium binding site of Aedes aegypti HAX1 parvalbumin-like domain modeled on the template of parvalbumin (PDB: 1RJP). A. N-terminal EF-hand motif - critical residues marked in blue; B. C-terminal EF-hand motif - critical residues likely to be involved in formation of calcium binding module were shown in red. [file 1756-0500-3-197-S1.PDF]
